# Tailoring Polymeric Scaffolds with *Buddleja globosa* Extract for Dual Antimicrobial and Biocompatible Wound Healing Applications

**DOI:** 10.3390/molecules30112428

**Published:** 2025-05-31

**Authors:** Ricardo Ceriani, Daniel A. Cherif-Pino, Pamela Pérez-Basáez, Marcela Escobar, Patricio Leyton, Caroline R. Weinstein-Oppenheimer, Daniel F. Moraga-Espinoza, Tania F. Bahamondez-Canas

**Affiliations:** 1 Escuela de Química y Farmacia, Facultad de Farmacia, Universidad de Valparaíso, Valparaiso 2340000, Chile; ricardo.ceriani@uv.cl (R.C.); marcela.escobar@uv.cl (M.E.); caroline.weinstein@uv.cl (C.R.W.-O.); 2 Centro para la Investigación Traslacional en Neurofarmacología (CITNE), Universidad de Valparaíso, Valparaiso 2340000, Chile; 3 Instituto de Química, Pontificia Universidad Católica de Valparaíso, Valparaiso 2340000, Chile; patricio.leyton@pucv.cl; 4 Centro de Investigación, Desarrollo e Innovación de Productos Bioactivos (CInBIO), Universidad de Valparaíso, Valparaiso 2340000, Chile

**Keywords:** scaffolds, natural extract, wound healing, antimicrobial, biocompatibility

## Abstract

Integrating traditional herbal extracts into modern biomaterials offers a promising route for advanced wound care. A standardized *Buddleja globosa* Hope extract (BG-126), recognized for its therapeutic value, was incorporated into polymeric scaffolds with variable composition to explore their potential in promoting wound healing and controlling infections. This work aimed to identify the polymeric composition of a scaffold with BG-126 that maximizes its compatibility and antimicrobial properties. Scaffolds were developed by lyophilization using a Box–Behnken design (BBD) with chitosan, hyaluronic acid, and gelatin content as study factors. Thirteen scaffold formulations were tested for their antimicrobial activity against *Staphylococcus aureus* and *Pseudomonas aeruginosa*, including biofilm forms, as well as for their biocompatibility with normal human fibroblasts. Structural and physical properties, such as the moisture content and swelling capacity, were evaluated. The best-performing scaffold was analyzed using Raman spectroscopy. The chitosan content was strongly associated with antimicrobial efficacy, while gelatin enhanced fibroblast compatibility (R^2^ ≥ 0.9). No correlations were identified between the polymeric content and biofilm inhibition or physical properties. BG-126-loaded scaffolds reduced planktonic and biofilm proliferation and improved fibroblast compatibility compared to the control scaffold (without BG-126). The results support the rational design of botanical-loaded scaffolds with targeted properties for wound healing.

## 1. Introduction

Wounds trigger a physiological healing process to recover the structural integrity of the skin barrier within a proper time and order. Those that fail to heal in this orderly and timely manner are known as chronic wounds. These wounds can remain unhealed for months or even recur due to different metabolic, nutritional, and local factors [1]. Infections are one of the local factors that disrupt the healing process and can lead to severe complications such as septicemia and limb amputation. On the other hand, chronic wounds are at high risk of infection due to their extended duration. Therefore, infection control is of utmost importance to prevent entering this vicious cycle. Infection control is achieved with frequent cleaning, on-time debridement, and protecting the exposed tissue with adequate wound dressings. The integrated wound care approach has shown promising results in decreasing the percentage of lower limb amputations from 15% to 6% in developed countries [2,3]. Still, these statistics are not applicable globally, and there is an important window of opportunity to improve wound healing.

One aspect that deserves attention is the prevention of biofilm formation. Biofilms are microbial communities encased within a self-secreted protective extracellular substance [4]. Consequently, biofilms are highly resistant and, once established, are extremely hard to eradicate. Biofilms are present in almost all chronic wounds, contributing to the chronic state of inflammation that characterizes these wounds [5,6]. However, using traditional antibiotics has disadvantages, including the risk of developing resistance, but also of cytotoxic effects on the fragile tissue of the wound [7,8,9]. Ciprofloxacin-loaded matrices made of chitosan and hyaluronic acid showed cytotoxic effects on dermal fibroblasts [10]. Indeed, these disadvantages have guided researchers toward natural extracts, with increasing research into incorporating herbal constituents into wound dressings [11]. *Buddleja globosa* (BG) is a native plant from Chile and Argentina that is traditionally used as a healing promoting agent to treat gastric and skin ulcers, with increasing scientific evidence of its properties [12,13]. Extracts of this plant have been shown to induce fibroblast proliferation [14] and reduce inflammation with analgesic properties [15]. Additionally, the antimicrobial activity of *B. globosa* constituents, such as thymol [16], quercetin [17], and carvacrol [18,19], has been reported against different bacterial and fungal species, including *Staphylococcus aureus* and *Pseudomonas aeruginosa,* the most prevalent pathogens isolated from complex wounds growing as biofilms [20]. A standardized hydroalcoholic extract of *B. globosa* Hope (BG-126) was shown to improve urinary tract infections [21], and its antimicrobial properties against *P. aeruginosa* have been recently reported [22].

Biodegradable scaffolds (bioresorbable dressings) comprising natural polymers have been shown to accelerate the healing process by supporting fibroblast proliferation, representing a milestone for wound care. These scaffolds can inactivate detrimental factors such as proteases and free radicals [23]. Since these scaffolds are meant to stay and integrate within the wound during healing, they represent an excellent platform for the topical delivery of therapeutic compounds with healing and antimicrobial properties by degradation or erosion [24]. Scaffolds consisting of chitosan, gelatin, and hyaluronic acid have shown the capacity to induce proliferation of fibroblasts and keratinocytes, expression of transforming growth factor beta-3 (TGFß3), and regeneration, among other factors [25,26,27]. These polymers are frequently used in scaffolds and are regarded as biocompatible materials [28,29]. Chitosan can present mild cytotoxicity when tested as solutions, but no toxicity has been reported when tested as carriers or scaffolds in vitro [30,31] and in vivo [32,33,34]. Gelatin compatibility may be affected by crosslinkers that are required to improve its physical properties. However, crosslinked gelatin with *N*-(3-dimethylaminopropyl)-*N*’-ethylcarbodiimide (EDC) and microbial transglutaminase has shown good compatibility in vivo [35]. Chitosan is a great candidate among these polymers due to its inherent antimicrobial activity. Chitosan is a natural polymer with a broad antimicrobial spectrum against bacteria and fungi, but low toxicity to mammalian cells [36,37]. Hyaluronic acid also shows anti-adhesive and antimicrobial properties [38,39] with dose-dependent effects reported against different species, including *P. aeruginosa* [40].

Despite the known antimicrobial properties of some polymers, these properties are rarely considered among the optimizable responses during the manufacturing of these wound healing scaffolds, which routinely focus on their physical properties and compatibility. Therefore, in this work, we aimed to study the effect of different polymeric contents on the antimicrobial properties of BG-126-loaded scaffolds against clinically relevant pathogens, *P. aeruginosa* and *S. aureus*. Despite the increasing evidence of the wound healing properties of *B. globosa* extracts, there are few commercial products available for their topical administration. Unlike existing semisolid formulations for *B. globosa* extract, our approach could offer the potential to simplify dosing and minimize discomfort during application.

## 2. Results

### 2.1. Scaffolds Manufacturing

We used a Box–Behnken Design (BBD) method to study the influence of the scaffolds’ polymeric composition on their physical and biological properties. Scaffolds with variable contents of three polymers (chitosan, hyaluronic acid, and gelatin) and a fixed content of a standardized herbal extract (BG-126) were designed and prepared according to Table 1. Each scaffold consists of a combination of three polymers at three different levels, low (−), central (0), and high (+), as shown in the pattern column. These levels were 1.9–3.8–5.7 mL for chitosan, 0.95–1.9–2.85 mL for hyaluronic acid, and 10.45–13.3–16.15 mL for gelatin content, respectively, as volumes from the stock solutions described in the Methods section. S1* was added to the design as a control scaffold and represents the only scaffold without the BG-126 extract.

### 2.2. Physical Characterization of the Scaffolds

**Figure 1:**
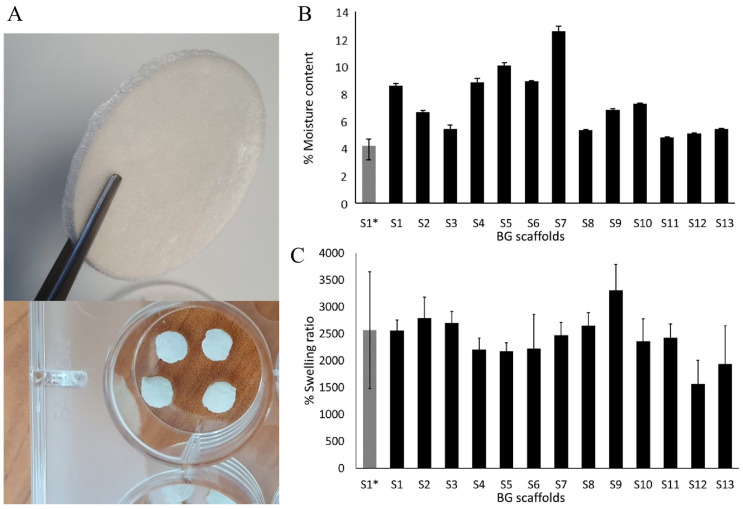
The moisture content and swelling behavior of the scaffolds. (**A**) A representative scaffold after lyophilization and round pieces of the scaffolds used for in vitro evaluation in fibroblasts and bacterial cultures. (**B**) The moisture content of the scaffolds, and (**C**) swelling behavior. The results are expressed as mean ± SD.

The scaffolds had a uniform aspect despite their polymeric composition and low density (0.02 g/mL), with an average weight of 0.3 g and 11.9 cm^3^ of volume (7.1 mm diameter × 3 mm height average) (Figure 1A). Neither the moisture content, the swelling ratio, nor the pore size of the scaffolds correlated with the polymeric composition when modeled by surface response methodology (R^2^ < 0.9).

The analysis after lyophilization showed a variable moisture content that was within 3.7% for S1* (control scaffold, without BG-126 extract) and 12.7% for S7 (Figure 1B). Only five scaffolds had residual moisture contents similar to S1* (S3, S8, and S11 to S13 within 4 and 5%). After 24 h of being submerged in PBS, the % swelling ratio was determined gravimetrically. All the scaffolds performed similarly, with a % swelling around 1500 and 3000%, and the lowest swelling was observed in S12. Overall, the scaffolds showed a % swelling like that of the referential scaffold (S1*), around 2500% in weight (Figure 1C).

**Figure 2:**
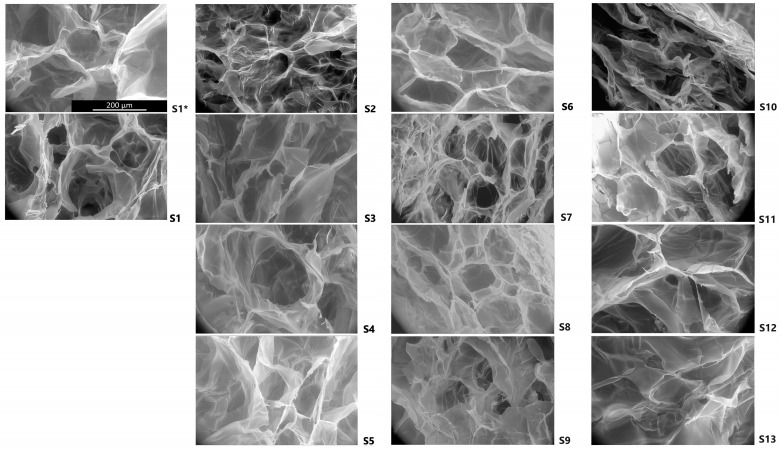
FESEM micrographs of the scaffolds. The image shows the morphology of the transversal cuts of representative scaffolds obtained at 650× magnification. The scale bar in S1* represents 200 µm and is applicable to all scaffolds’ images.

Scanning electron microscopy (SEM) images of transversal cuts of the scaffolds confirmed the achievement of a highly porous material (Figure 2). The pores had heterogeneous shapes and sizes, with some interconnections. The average pore diameter of all scaffolds was 111 µm, ranging from 70 to 154 µm. Table 2 shows the calculated pore diameters determined by Trainable Weka Segmentation (TWS) analysis. S11, S13, S1*, S7, and S10 had average diameters below 100 µm and S4, and S2 had pores above 150 µm of diameter.

### 2.3. Effect of BG-126 Scaffolds on Bacterial Proliferation and Adhesion

**Figure 3:**
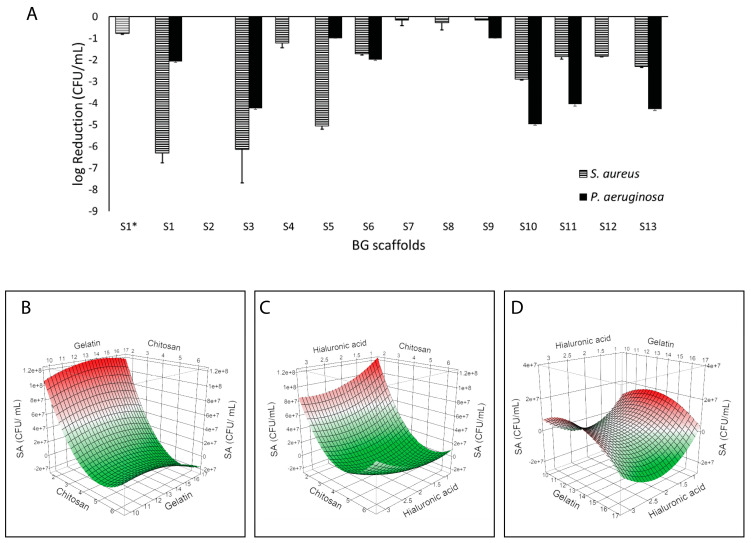
Inhibitory properties of the BG-126 scaffolds. The scaffolds were tested on *P. aeruginosa* (black bars) and *S. aureus* (hatched bars). S1* does not contain BG-126 (**A**). The results are expressed as a logarithmic reduction mean ± SD. Surface response plots of the inhibitory effect of BG-126 scaffolds on *S. aureus* showing the relation between chitosan and gelatin with bacterial viability (**B**), chitosan and hyaluronic acid (**C**), and gelatin and hyaluronic acid (**D**). The color gradient shows the highest inhibitory effect in green, and the lowest is in red.

BG-126 scaffolds were developed according to the BBD and were named S1* (central level scaffold without BG-126 extract) and S1 to S13 with fixed BG-126 and different polymeric contents. Firstly, we could observe a difference between the effect of S1* (without BG-126) and S1 (with BG-126). S1 decreased bacterial survival, especially on *S. aureus*, followed by S3 and S5, while S3, S10, S11, and S13 had the highest inhibitory effects on *P. aeruginosa* (Figure 3A). Supplementary Figure S1A,B show the surviving colonies in agar after plating the supernatants by the drop plate method.

When modeling bacterial viability (CFU/mL) as a response by a surface response methodology, we could identify a correlation between *S. aureus* viability and the polymeric content of the BG-126 scaffolds (R^2^ = 0.98; *p* = 0.0012) (Figure 3B–D). Specifically, the chitosan content was strongly associated with lower *S. aureus* viability (*p* = 0.0001). When comparing the two variables, no interactions were determined. Therefore, the inhibitory effect was mainly given by chitosan. No correlation was identified between *P. aeruginosa* viability and the polymeric content.

**Figure 4:**
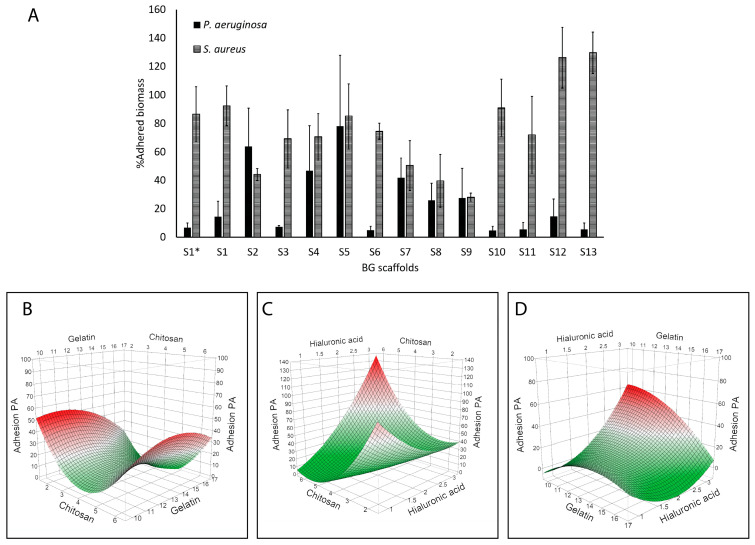
Antibiofilm effects of the BG-126 scaffolds. Biomass of *P. aeruginosa* (black bars) and *S. aureus* (hatched bars) adhered to the wells of the plate after incubation with the scaffolds (**A**). S1* does not contain BG-126. The results are expressed as % adhered biomass ± SD. Relation between chitosan and gelatin with bacterial adhesion (**B**), chitosan and hyaluronic acid (**C**), and gelatin and hyaluronic acid (**D**). The color gradient shows, in green, the lowest adhesion, and in red, the highest.

Figure 4 shows the effect of the scaffolds on the amount of adhered bacterial biomass quantified by crystal violet. S1* and S1 showed similar results, and overall, the scaffolds showed higher inhibition of *P. aeruginosa* adhesion (reaching values below 10% with S3, S6, S10, S11, and S13) as compared to *S. aureus* (S9 near 30%) (Figure 4A). After modeling the results, we found a correlation with *P. aeruginosa* adhesion (R^2^ = 0.93; *p* = 0.0195). Figure 4B–D show the surface response plots on *P. aeruginosa* adhesion, and we could identify a correlation between this response and chitosan (*p* = 0.0152) and the hyaluronic acid content (*p* = 0.0456), as well as with the association of both polymers (*p* = 0.0034). Indeed, the highest reduction in *P. aeruginosa* adhesion (i.e., prevention of biofilm formation) was achieved at the highest chitosan content and the lowest hyaluronic acid content. No correlation was observed with *S. aureus* adhesion.

### 2.4. Inhibitory Properties of BG Scaffolds on Bacterial Biofilms

**Figure 5:**
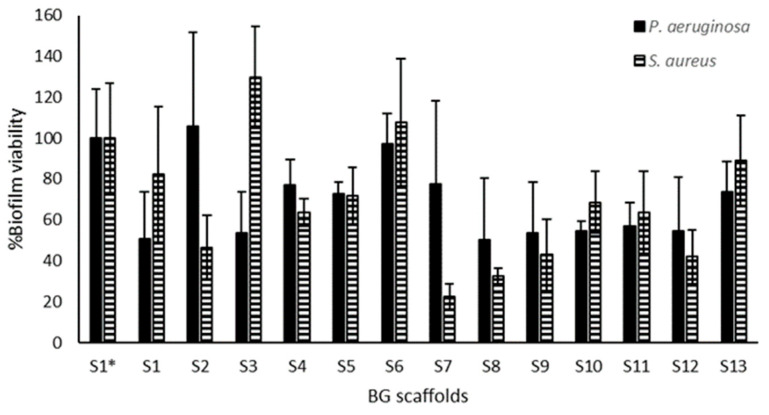
Biofilm inhibitory activity of the BG scaffolds. Scaffold pieces were placed above preformed biofilms of *P. aeruginosa* (black bars) and *S. aureus* (hatched bars), and the viability of these treated biofilms was quantified by a metabolic assay. S1 to S13 contain BG-126, and S1* does not contain BG-126. The results are expressed as % biofilm viability ± SD.

Figure 5 shows that the scaffolds had slightly higher inhibitory activity on *S. aureus* biofilms than the *P. aeruginosa* biofilms, and S7 had the highest inhibition on *S. aureus* biofilms. In general, the highest biofilm inhibition was around 50 to 80% for S9 to S12 for both species. However, when modeling biofilm inhibition with respect to the polymeric content, no correlation was observed for any of the species tested.

### 2.5. Compatibility of Scaffolds

**Figure 6:**
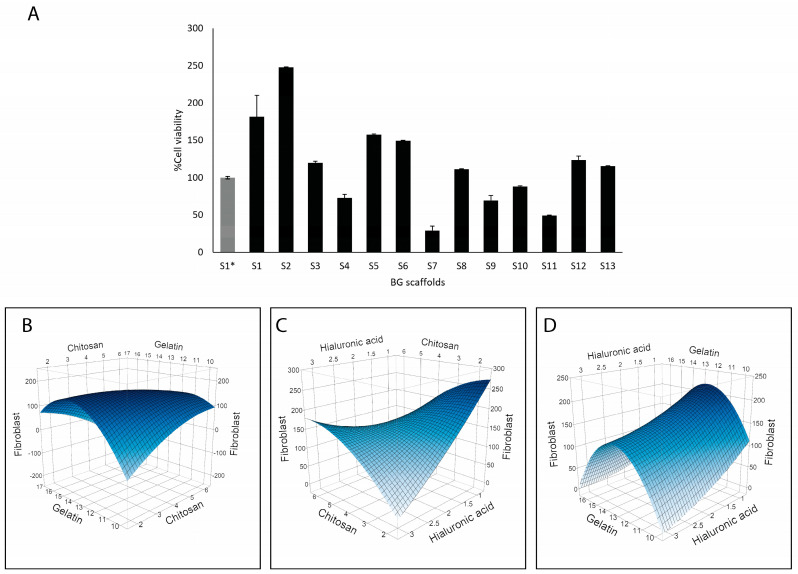
Compatibility of the BG-126 scaffolds with human dermal fibroblasts. (**A**) Cell viability within the scaffolds was quantified after 3 days of incubation. S1* does not contain BG-126. The results are expressed as % cell viability ± SD with respect to S1*. Relation between chitosan and gelatin with fibroblast viability (**B**), chitosan and hyaluronic acid (**C**), and gelatin and hyaluronic acid (**D**). The color gradient shows, in dark blue, the highest viability, and in light blue, the lowest.

BG scaffolds are meant to improve wound healing by acting as a substrate for cell proliferation. Therefore, the viability of human dermal fibroblasts growing on the scaffolds was determined after 3 days (Figure 6). Around half of the prototype scaffolds resulted in enhanced compatibility compared to S1*, with the highest viability obtained with S2 and S1 (Figure 6A). Modeling these results showed that gelatin was strongly associated with fibroblast viability (*p* = 0.0054) (Figure 6B–D). On the other hand, hyaluronic acid correlated negatively with fibroblast viability (*p* = 0.0353). Additionally, interactions of gelatin with chitosan (*p* = 0.0442) and gelatin with hyaluronic acid (*p* = 0.0190) had significant effects on fibroblast viability, indicating that the highest viability was achieved with higher levels of gelatin and lower levels of chitosan and hyaluronic acid.

When analyzing the correlation of the scaffold’s composition with the antimicrobial properties (Figure 3 and Figure 4) and compatibility with fibroblasts (Figure 6), we could identify S3 as the scaffold with the best balance between antimicrobial activity and compatibility. This scaffold has more chitosan, less hyaluronic acid, and equal amounts of gelatin compared to S1* (see composition in Table 1).

### 2.6. Molecular Interactions and Content Uniformity of Scaffolds

**Figure 7:**
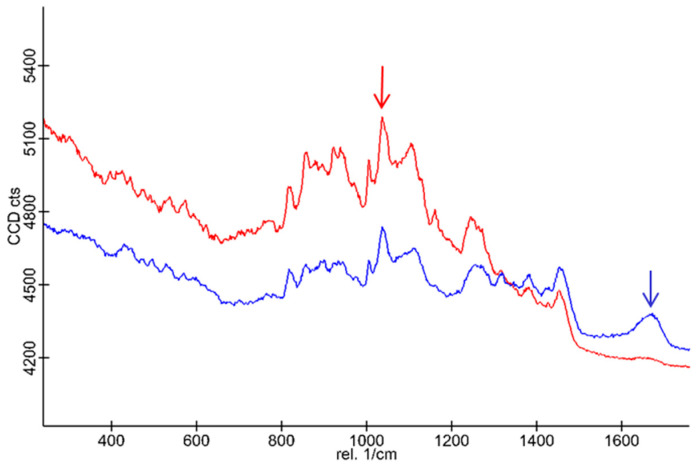
Raman spectra of the scaffolds S1* and S3. The S1* spectrum (in red) and the S3 spectrum (in blue) show the characteristic bands of the polymeric scaffold (red arrow) and the BG-126 extract (blue arrow).

Scaffolds S1* (without BG-126; in red) and S3’s (in blue) Raman spectra are shown in Figure 7. Characteristic broad bands are observed for chitosan at 1458, 1383, 1245, 1162, 1108, 1041 (red arrow), and 1007 cm^−1^ and between 1000 and 800 cm^−1^ with medium intensity. The S1* and S3 samples have similar spectral profiles except for the notable growth and displacement of a band centered at 1670 cm^−1^ (blue arrow). Likewise, the decrease in intensity of the bands at 1162 and 923 cm^−1^ is also observed, which disappears in the spectral background. In this context, there are more spectral characteristics that decrease in relative intensity than those that increase it. No large shifts in the wavenumber are observed, which is consistent with physical adsorption, also accompanied by a broadening of the bands in the S3 spectrum, which accounts for interactions between the polymers and the complex mixture of the BG-126 extract.

**Figure 8:**
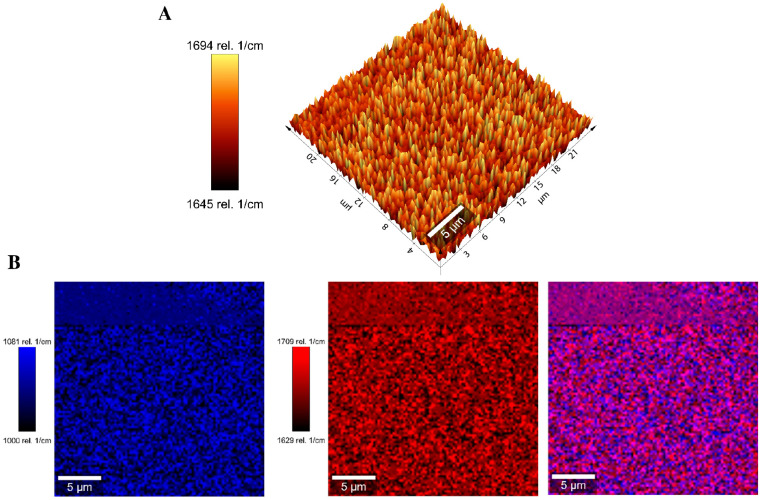
Signal tracking by Raman spectroscopy of the scaffolds S1* and S3. (**A**) Signal tracking of the characteristic band of BG-126 extract at 1670 cm^−1^ in S3; (**B**) distribution of the BG-126 extract (blue) on the polymeric scaffold (red) and the combined distribution (purple) from the spectra recorded in an area of 25 × 25 µm.

The band at 1670 cm^−1^ was selected to measure the homogeneity of the BG-126 extract distribution in scaffold S3 through signal tracking (Figure 8A). Thymol, carvacrol, catechin, and quercetin (some relevant BG-126 components) spectra were also obtained, and the signal at 1670 cm^−1^ was attributed to quercetin. This signaling allowed us to identify a homogeneous distribution of BG-126 in the scaffold sample and was comparable throughout the scanning area. This distribution is also observed when combining the tracked signals of the scaffold (1041 cm^−1^, in blue) and the BG-126 extract (1670 cm^−1^, in red) (Figure 8B).

## 3. Discussion

We developed prototype scaffolds to identify the best formulation based on their bioactivity using a Design of Experiments (DoE) approach. DoE has gained space in pharmaceutical formulation development as an alternative to the traditional, but inefficient, trial-and-error approach. DoE allows for studying the influence of multivariable changes (i.e., formulation and processing variables) on certain responses, and these responses are commonly physical characteristics of the formulations [41,42]. We aimed to optimize the composition of the scaffolds to achieve enhanced compatibility and antimicrobial activity, both biological properties. To our knowledge, applying a DoE methodology to optimize biological properties is uncommon. Dellaquila et al. applied a full factorial design to develop hydroxyapatite and collagen scaffolds and tested the physical properties of the scaffolds, such as the porosity, swelling ratio, and degradation rate [43]. Similarly, Shirzad et al. used a central composite design to study the physical and mechanical properties of their polymethyl methacrylate scaffolds for bone tissue [44]. In our work, we tested the antimicrobial properties and compatibility, showing the applicability of this tool to design scaffolds for tissue engineering rationally. While testing biological responses involves their inherent variability, as a screening stage of the DoE, we were able to obtain results that correlated with literature reports, such as the antimicrobial properties of chitosan [45].

### 3.1. Role of Chitosan on the Antimicrobial Effect of the Scaffolds

We could identify the polymeric composition correlated with enhanced antimicrobial activity when studying bacterial inhibition (Figure 3) and adhesion (Figure 4). S3 showed enhanced activity, having less hyaluronic acid and higher chitosan, and similar compatibility with respect to the referential scaffold (S1*). We found that the chitosan content correlated with reduced *S. aureus* viability (Figure 3) and antibiofilm activity on *P. aeruginosa* (Figure 4). Though both species are among the most commonly isolated bacteria from chronic wounds, Fazli et al. reported that *S. aureus* is located near the wound’s surface, while *P. aeruginosa* is found in deeper regions near the tissue [46], indicating that *P. aeruginosa* may play a more important role in the chronic inflammation and subsequent delayed healing of these wounds. Infection control is of utmost importance in wound management. Therefore, a dressing for chronic wounds should prevent bacterial proliferation, adhesion, and biofilm formation, and ideally, eradicate pre-established biofilms. Biofilms, due to their adherent nature, are hard to remove, and in wounds, debridement is an important part of wound care that contributes to wound healing [47]. On the other hand, planktonic bacteria (free-floating) can be removed with frequent rinsing. Therefore, polymers with antibiofilm activity are ideal scaffold components to prevent biofilm infections and promote wound healing. Between *S. aureus* and *P. aeruginosa*, the latter biofilms are associated with more complications and severe delayed healing in diabetic wounds and venous leg ulcers [46,48,49,50].

Chitosan adheres to bacterial cell membranes, leading to the leakage of cell components [45]. In our study, we used chitosan with a molecular weight (MW) of 250–350 kDa and with 98% deacetylation. Omura et al. and Másson studied the effect of these properties on the antimicrobial activity of chitosan against different bacterial species (including *S. aureus* and *P. aeruginosa*) and found that MWs 300–400 kDa and deacetylations near to 100%, similar to those used in our study, had the highest antimicrobial activity compared to MW below 1 kDa and deacetylation below 57% [51,52]. These results suggest that the number of free amine groups in chitosan determines the antimicrobial properties that depend on both MW and deacetylation degree. Finally, Tao et al. studied the inhibitory effect of chitosan on both bacterial species studied herein and found that *S. aureus* was more sensitive to chitosan than *P. aeruginosa* [45].

It is important to emphasize that most studies on the antimicrobial activity of chitosan are based on the broth microdilution method using chitosan solutions, which differs from our scaffolds, which it is in a solid state and chemically crosslinked with gelatin and HA. Nevertheless, Wu et al. developed films made of polymeric blends, PVA/chitosan films, that significantly reduced *P. aeruginosa* adhesion and subsequently biofilm formation [53]. Liu et al. also found that either cationic or anionic chitosan can prevent *P. aeruginosa* biofilm formation [54]. These results correlated with our findings on planktonic bacterial proliferation and adhesion, providing an endorsement for our DoE approach.

### 3.2. Effect of Scaffolds on Fibroblast Viability

We tested scaffolds’ cytocompatibility with human dermal fibroblasts (Figure 6). Fibroblasts are the main cells in wound healing, responsible for collagen production, wound contraction, tissue remodeling, and secretion of growth factors and cytokines that promote tissue regeneration [55,56]. Because of this role, fibroblasts have also been included in commercial products for burns and ulcers [56]. Therefore, growing these cells in scaffolds is commonly used to determine the compatibility for tissue regeneration, using metabolic assays for cell viability, cell proliferation, or histology analysis to visualize the cells in the scaffolds [57].

We used resazurin, a common reagent for cell viability determination. Live cells reduce resazurin, a blue dye, to produce pink, fluorescent resorufin that can be quantified by spectrophotometry. This method has shown good linearity between the signal and cell number in cells grown within scaffolds [58]. In our work, the results are expressed as a % viability with respect to the referential scaffold (S1*) in Figure 6. Most of the formulations performed similarly or better than S1*. Scaffold S2 (248%) followed by S1 (182%) promoted the highest fibroblast viability, and the gelatin content correlated with enhanced fibroblast viability (Figure 6). However, when looking at the optimization potential of this response, the options given by the model are increasing the gelatin composition while decreasing the chitosan and hyaluronic acid contents. Accordingly, S2 had a lower content of chitosan and hyaluronic acid plus BG-126, which could also contribute to the enhanced viability as discussed above. Gelatin is a compatible and biodegradable polymer that has shown comparable results in terms of cell adhesion and proliferation to collagen [59], and therefore, it was expected to find this effect. However, a decreasing chitosan content will be detrimental to the antimicrobial properties. Barton and collaborators tested bacterial adhesion on different polymeric implants and found that polymers exposed to hyaluronic acid had enhanced adhesion of *E. coli* and *P. aeruginosa* [60]. Since our results showed that hyaluronic acid correlated with a reduced viability of fibroblasts and with higher *P. aeruginosa* adhesion, we might have room to optimize the hyaluronic acid content.

According to these results and our design, the leading scaffold was S3, consisting of the highest levels of chitosan, the central level of gelatin, and the lowest level of hyaluronic acid studied. This scaffold had a residual moisture content and swelling behavior similar to the control scaffold (S1*). Considering that hyaluronic acid is recognized by its relevant properties for tissue engineering [61], it may still be beneficial for wound healing based on activities not included in this study, such as keratinocyte viability or the release of growth factors. Therefore, scaffold S3 could be considered the lead candidate with optimized polymeric composition.

### 3.3. Effect of BG-126 on the Antimicrobial Properties and Compatibility of the Scaffolds

We developed scaffolds loaded with a fixed concentration of *B. globosa* extract (BG-126) and focused on optimizing the polymeric composition. We found that the addition of BG-126 provided enhanced antimicrobial properties and compatibility. Since all the scaffolds have different polymeric compositions, these observations come from the comparison between S1 (with BG-126) and S1* (control scaffold, without BG-126), with the same polymeric composition (all polymer content at the central level). S1 reached a 6-log reduction on planktonic *S. aureus* and about a 2-log reduction in *P. aeruginosa,* compared to a small effect in *S. aureus* (−1 log) and no effect in *P. aeruginosa* with S1* (Figure 3). Therefore, based on the modeling, this small effect observed on *S. aureus* could be attributed to chitosan in S1*, and the enhanced effect observed with S1 could be attributed to the combination of chitosan plus BG-126.

Here, we used a low concentration of BG-126 to mainly focus on optimizing the polymeric content of the scaffolds. In a previous study, BG-126 showed significant inhibitory activity on *P. aeruginosa* proliferation at concentrations equal to or above 128 µg/mL total phenol content, expressed as catechin (or 64 µg/mL verbascoside) [22]. Therefore, this inhibitory effect on *P. aeruginosa* could not be observed here since we used lower concentrations. Avila et al. reported that verbascoside has inhibitory activity on *S. aureus* by targeting protein synthesis [62].

Although the scaffolds exhibited an antimicrobial effect against planktonic bacteria that correlated with chitosan concentration (Figure 3 and Figure 4), this correlation was not observed in established biofilms. Chitosan induces the hydrolysis of the peptidoglycans of the bacterial wall, and this effect appears to be stronger on Gram-positive bacteria, such as *S. aureus*, than on Gram-negative bacteria like *P. aeruginosa* [63,64]. In established biofilms, where an extracellular substance physically protects the bacteria, the surface-level action of chitosan might be limited [6]. Therefore, the use of chitosan has primarily focused on either preventing biofilm formation or developing nanoformulations or derivatives to enhance its activity against biofilms. [65,66]. Still, the action of the scaffolds on pre-established biofilms could be optimized based on the leading scaffold S3 by testing new factors such as the content of the bioactive compounds. Figure 5 shows that S1 (with BG-126) had enhanced biofilm inhibitory activity compared to S1* (without BG-126), decreasing *P. aeruginosa* biofilm viability by about 50% and *S. aureus* by 20%. Therefore, the effect of different concentrations of BG-126 on the optimized scaffold on biofilm inhibition and formation remains to be investigated, as well as the mechanisms of release of BG-126 components from the optimized scaffolds.

When comparing S1 to S1*, we also observed an enhanced viability of fibroblasts (Figure 6). *B. globosa* extracts have shown a slight increase in fibroblast growth [14] but also cytotoxic effects on Chinese hamster ovary (CHO-K1) cells [15]. Therefore, increasing its content on the scaffolds should be carefully and further assessed in relevant skin cell lines such as fibroblasts and keratinocytes.

### 3.4. Physical Properties of the Scaffolds

No correlation was found between the polymeric composition and the swelling ratio, moisture content, or pore sizes (Figure 1B,C and Table 1). The swelling ratio of all formulations was similar (Figure 1C). When calculating the mass of each polymer in the final scaffold (Table 1), gelatin ranges from approximately 50% to 80%, chitosan ranges from 20% to 50%, and hyaluronic acid accounts for less than 1% of the scaffold mass. Since gelatin is the main constituent in all the scaffolds and all the scaffolds were subjected to the same crosslinking treatment (EDC HCl and NHS in MES buffer), it could be expected that the main factor influencing these properties would be gelatin. In our study, the scaffolds with the highest and lowest swelling ratios were S9 and S12, respectively, with the highest (80%) and the lowest (50%) gelatin contents. These results correlated with the study of Nagahama et al., in which the authors developed hydrogel membranes made of different ratios of gelatin/chitosan [67] and found that the membrane with 70:30 gelatin/chitosan (the highest ratio tested) had the highest swelling ratio. Therefore, it could be suggested that we could not find a correlation with this physical property because the proportion of gelatin in the scaffolds in our modeling was set too high with respect to the other two polymers.

The scaffolds’ pore sizes were highly variable (Figure 2). Though our study could not identify a correlation with the scaffolds’ components, it can be observed that scaffolds with lower viability (S7, S9, and S11, Figure 6) had smaller pore sizes (near or below 100 µm, Table 2). Mandal et al. studied the effect of pore size, porosity, and connectivity and found that scaffolds with pores of 200–250 µm and those with 80–100 µm had similar cell proliferation, but higher interconnectivities, even with smaller sizes, allowed maximum cell migration [68]. They also found that scaffold pore sizes were larger when using higher freezing temperatures before lyophilization (−20 °C vs.−80 °C). In our study, we started a gradual freezing at 2–8 °C, which may lead to higher or more variable pore sizes.

Finally, we conducted Raman spectroscopy of S1* and the best-performing scaffolds S3. Zajac et al. previously reported the spectral assignment for Figure 7 in the literature. [69] Moreover, in general, it corresponds to a set of coupled vibrations of the polymeric skeleton (Supplementary Table S1). The band at 1670 cm^−1^ in the S3 spectra is assigned to the carbonyl group, and it could be associated with a flavone fragment [70]. Additionally, the decrease in intensity of the bands at 1162 and 923 cm^−1^ observed in S3 is probably due to the presence of the BG-126 extract in the interstices of the scaffold’s polymer matrix. When using signal tracking of characteristic bands of S3 and S1*, a homogeneous distribution of the BG-126 extract in the scaffold’s polymeric matrix was confirmed (Figure 8), which indicates that a uniform dosing of BG-126 was achieved in the scaffold. Also, the broadening of the bands in the S3 spectrum (Figure 8B, in blue) reflects interactions between the polymers and the complex mixture of the BG-126 extract. This broadening in the bands could represent the non-covalent interactions reported in the literature between phenolic compounds (present in BG-126) and gelatin [71,72]. These interactions may modulate the release profile of BG-126, suggesting that the scaffolds’ degradation, rather than simple diffusion, plays a more prominent role in its release mechanism [73]. Here, we tested a fixed extract concentration, though the effect of the extract on the scaffolds’ physical and mechanical properties, as well as the release mechanism of BG-126, could be investigated further.

## 4. Materials and Methods

### 4.1. Materials

Chitosan, from crab shells, was obtained from Quitoquimica (Coronel, Chile), having a MW range of 250–350 kDa and a 98% deacetylation degree. Sodium hyaluronate (MW range 100 to 1800 kDa) was purchased from Lifecore Biomedical (Chaska, MN, USA). Gelatin from bovine skin type B (MW range 50–100 kDa), *N*-hydroxysuccinimide (NHS), *N*-(3-dimethylaminopropyl)-*N*’-ethylcarbodiimide hydrochloride (EDC HCl), MES hydrate, fluorescein diacetate (FDA), resazurin sodium salt, bovine plasma, thymol, carvacrol, catechin, and quercetin were provided by Sigma Aldrich (St. Louis, MO, USA). Other reagents were of analytical grade. A standardized hydroalcoholic extract of *Buddleja globosa* Hope (BG-126) was kindly donated by Laboratorios Ximena Polanco (Santiago, Chile). This extract was previously characterized by our group and contains 7378 µg/mL total phenolic content (expressed as catechin) and 3696 µg/mL verbascoside [22].

### 4.2. Design of Experiment (DoE) to Optimize the Scaffolds’ Polymeric Content

A DoE for prototype development was performed using the software JMP 10.0.0 (SAS Institute Inc., Cary, NC, USA). Among the response surface methodologies, the Box–Behnken design (BBD) was selected since it results in a minimal number of experimental runs and it avoids extreme axial points [74]. Here, a three-level BBD was defined for three different factors (chitosan, gelatin, and hyaluronic acid content) with respect to the referential polymeric scaffold reported by Enrione et al. [75]. This scaffold served as the central level of the DoE (S1), which was randomly included in triplicate within the manufacturing process as a control of the DoE. The remaining scaffolds (S2 to S13) were manufactured once.

### 4.3. Scaffolds Development

A 2% *w*/*v* chitosan stock solution was prepared in 1% *v*/*v* acetic acid at 50 °C. Gelatin (1% *w*/*v*) and hyaluronic acid (0.01% *w*/*v*) stock solutions were prepared in distilled water at 50 °C. BG-126 [22] was diluted in 50% *v*/*v* ethanol to obtain a final concentration of 1 µg/mL of verbascoside, 6.5 µg/mL of total flavonoid content expressed as quercetin, and 2 µg/mL of total phenol content expressed as catechin in the polymeric solution. Then, the polymeric solutions (Table 1) and the diluted BG-126 were combined to obtain different proportions of polymers and a fixed concentration of BG-126. The mixtures were poured within 90 mm sterile Petri dishes that were gradually frozen by storing them at 2 to 8 °C, −20 °C, and −80 °C for 24 h each until lyophilization for 48 h (INOFD-18PU, Qingdao Innova Bio-meditech Co., Ltd., Qingdao, China).

After lyophilization, the scaffolds were crosslinked. Briefly, the scaffolds were soaked in 20 mL of a 1:10 mixture of 0.5 M MES buffer and absolute ethanol for 1 h, flipping the scaffolds every 15 min. Then, the excess of the solution was discarded. A crosslinking solution of 0.08 M NHS and 0.3 M EDC HCl was prepared in 0.5 M MES buffer and diluted 1:10 in absolute ethanol. The scaffolds were soaked in 20 mL of this solution for 2 h, flipping the scaffolds every 30 min. After discarding the excess crosslinking solution, the scaffolds were serially rinsed in gradients of ethanol (10 mL) for 30 min each, starting with absolute ethanol, followed by 70% *v*/*v* ethanol, 40% *v*/*v* ethanol, and distilled water. The scaffolds were placed in Petri dishes and frozen before lyophilization for 48 h. Finally, the scaffolds were sterilized by exposure to UV radiation for 30 min on each side. Then, they were cut into circular pieces (6 mm diameter) for further testing. Their compatibility, swelling behavior, moisture content, and antimicrobial properties were defined as the responses of the DoE.

### 4.4. Physical Characterization of the Scaffolds

Scaffold pieces were loaded onto aluminum pans and heated to 160 °C for 10 min using a halogen moisture analyzer (HE53, Mettler Toledo, Leicester, UK), after confirming the adequacy of this drying time to achieve a plateau in the weight of the samples. The final weight was recorded to determine the % moisture content of the scaffolds. The swelling behavior was determined gravimetrically by soaking pieces of the scaffolds for 24 h in 1 mL of phosphate-buffered saline (PBS). The weight of the scaffold was recorded before (W_i_) and after soaking in PBS (W_f_), and the % swelling ratio was calculated as follows: % Swelling ratio = (W_f_ − W_i_)/W_i_ × 100.

Pore size was assessed by SEM. Micrographs of transversal cuts of each scaffold were obtained using a field emission scanning electron microscope (Quattro S, ThermoFisher Scientific Inc., Waltham, MA, USA) with a gaseous detector (GSED) in the environmental mode (ESEM). Pore size was determined with the software Fiji ImageJ 1.54f [76] using the TWS plugin [77]. TWS is based on machine learning, which enabled reproducible segmentation (i.e., boundary recognition) of the scaffold pores through an iterative training process involving manual pore recognition. The calculated areas were converted to circular diameters to ensure consistency.

### 4.5. Antimicrobial Properties of the Scaffolds

*Pseudomonas aeruginosa* (ATCC 27583) and *Staphylococcus aureus* (ATCC 27213) were provided by Microbiologics Inc. (St Cloud, MN, USA). Bacteria from frozen stocks were subcultured in agar overnight at 37 °C using Luria Bertani (LB) agar for *P. aeruginosa* and tryptic soy agar (TSA) for *S. aureus*. Then, 3 to 5 colonies were suspended and incubated overnight in LB for *P. aeruginosa* and TSB for *S. aureus* at 180 rpm and 37 °C. The suspensions were adjusted with a spectrophotometer at 625 nm to obtain 3 × 10^8^ CFU/mL (Synergy H1M, Agilent Technologies Inc., Santa Clara, CA, USA).

Scaffold pieces of 6 mm diameter were placed within 96-well plates. The adjusted suspension was 100× diluted in culture media (either LB or TSB) and poured over the scaffolds (200 µL per well). Then, the plates were incubated at 75 rpm and 37 °C for 24 h. After incubation, the supernatants were serially diluted in PBS in 10-fold dilutions before counting viable CFUs by the drop plate method [78] in LB (*P. aeruginosa*) or TSA plates (*S. aureus*). The results are reported as the logarithmic reduction in the number of surviving colonies (CFU/mL) with respect to the survival of the untreated control.

For bacterial adhesion, the supernatants and remaining scaffolds were discarded from the wells, and the plates were fully rinsed with distilled water. Methanol (200 µL) was used for 30 min to fix the biofilms to the wells before staining with a crystal violet solution (0.1% *w*/*v*) for 20 min. Then, the plates were rinsed and allowed to air dry overnight. Acetone (30% *v*/*v*) was used to dissolve the retained crystal violet, and the absorbance of this reagent was recorded at 590 nm. The results are reported as % adhered biomass with respect to the absorbance of the reference scaffold (S1*).

Biofilms of *P. aeruginosa* were grown as follows: 100 µL of the adjusted suspension (described above) was added to the wells of black flat-bottomed 96-well plates. After 1.5 h of incubation at 75 rpm and 37 °C, the supernatant was discarded, and the plates were rinsed twice with 100 µL of PBS. Then, 200 µL of LB broth was added to the wells, and the plates were sealed before a final incubation at 75 rpm and 37 °C for 24 h. For *S. aureus*, 200 µL of the adjusted suspension was added to the wells of black flat-bottomed 96-well plates and incubated statically at 37 °C for 24 h. After rinsing the biofilms with PBS, the scaffolds were carefully placed on top and covered with 200 µL of PBS before incubation at 37 °C for 24 h. After incubation, the supernatant and scaffolds were removed, and the viability assays were performed using FDA for *P. aeruginosa* and resazurin for *S. aureus* [79]. The results are reported as % biofilm viability with respect to the fluorescence recorded from the control group.

### 4.6. Compatibility with Human Dermal Fibroblasts

The pieces of scaffolds were conditioned for cell culture by soaking them in DMEM supplemented with fetal bovine serum (FBS) plus antibiotics (penicillin/streptomycin) at 37 °C and 5% CO_2_ for 24 h. Then, the scaffolds were placed within 96-well plates, loaded with 10,000 cells/well, and incubated under the same conditions for 3 days. After incubation, the scaffolds were carefully transferred to a new plate to perform a resazurin assay [80]. Each piece of scaffold was covered with 150 μL of resazurin (40 mg/L), followed by a 4 h incubation. The fluorescence of the reagents was recorded at λ 560 excitation/590 emission. The results are reported as % cell viability.

### 4.7. Raman Spectroscopy

The best-performing scaffold regarding biological properties (S3) was analyzed by Raman spectroscopy. Gelatin, chitosan, and BG-126 components (quercetin, catechin, carvacrol, and thymol) were included in the analysis as scaffold constituents. A fragment of the scaffold was placed on a glass slide, and the spectrum was subsequently recorded using a SNOM-Raman Witec Alpha 300 microscope equipped with a 785 nm excitation laser line and an electrically cooled CCD detector. The signal was calibrated using the silicon line at 520 cm^−1^, using a monocrystalline Si sheet and a 20× objective. The laser power on the samples was 2 mW. The resolution was set at 4 cm^−1^, and 10 scans were performed with an integration time of 1 s. The spectra were recorded in the range between 200 and 1800 cm^−1^. Finally, to track BG-126 extract in the scaffold, an area of 25 × 25 µm was scanned using 100 points per line and 100 lines per image with a scanning speed of 4.732 (s/line).

### 4.8. Statistical Analysis

The formulation design and evaluation of the scaffolds were performed using the DoE tool for Response Surface Design with three center points with the software JMP^®^ version 10.0.0 (SAS Institute Inc., Cary, NC, USA). The goodness-of-fit of the model (reported as R^2^) between the factors (the polymer content) and responses (the physical and biological properties of the scaffolds) was analyzed with this tool and reported as significant when *p* < 0.05.

## 5. Conclusions

We could determine the optimal polymeric content of chitosan, gelatin, and hyaluronic acid of the scaffolds loaded with a natural *Buddleja globosa* Hope extract (BG-126). Scaffolds with different polymeric contents were developed and modeled using a Box–Behnken design using surface response methodology. The chitosan content correlated with the inhibition of *S. aureus* and antibiofilm activity on *P. aeruginosa,* while gelatin correlated with fibroblasts’ viability. The addition of BG-126 further contributed to the *S. aureus* and *P. aeruginosa* planktonic and biofilm inhibition and improved compatibility. Raman spectroscopy confirmed a homogenous distribution of BG-126 in the scaffold through signal tracking. The polymeric composition of the scaffolds can be optimized to maximize the desired biological properties for wound healing, which can be improved by adding herbal extracts with antimicrobial and regenerative properties.

## Figures and Tables

**Table 1:** Composition of the scaffold generated by a Box–Behnken design.

Scaffold	Pattern	Chitosan (mL)	Hyaluronic Acid (mL)	Gelatin (mL)
S1* ^a^	000	3.8	1.9	13.3
S1 ^b^	000	3.8	1.9	13.3
S2 ^c^	−−0	1.9	0.95	13.3
S3	+−0	5.7	0.95	13.3
S4	0+−	3.8	2.85	10.45
S5	++0	5.7	2.85	13.3
S6	0−−	3.8	0.95	10.45
S7	−0−	1.9	1.9	10.45
S8	−0+	1.9	1.9	16.15
S9	−+0	1.9	2.85	13.3
S10	0−+	3.8	0.95	16.15
S11	0++	3.8	2.85	16.15
S12	000	3.8	1.9	13.3
S13	000	3.8	1.9	13.3

^a^ S1*: Central level scaffold without BG-126. ^b^ S1: Central level scaffold with BG-126, prepared in triplicate. ^c^ S2 to S13: Scaffolds at different levels with BG-126, prepared once.

**Table 2:** The pore diameters of the scaffolds obtained by TWS.

Scaffold	Mean Pore Diameter (µm)	Pore Diameter Range (µm)
S1*	81	16–229
S1	112	29–220
S2	154	74–216
S3	116	58–185
S4	152	84–219
S5	132	34–290
S6	140	56–314
S7	85	13–161
S8	121	10–283
S9	100	17–154
S10	93	5–266
S11	70	18–160
S12	116	17–160
S13	78	17–160

S1*: Scaffold with a polymeric content at the central level without BG-126. S1: Scaffold with a polymeric content at the central level with BG-126. S2 to S13: Scaffolds with different polymeric contents with BG-126.

## Data Availability

The original contributions presented in this study are included in the article/Supplementary Materials. Further inquiries can be directed to the corresponding authors.

## Supplementary Materials

The following supporting information can be downloaded at: https://www.mdpi.com/article/10.3390/molecules30112428/s1, Supplementary Figure S1A,B: Surviving colonies in agar after plating the supernatants by the drop plate method; Table S1: Vibrational assignment of scaffold S1* (without BG-126 extract).
