# Supplementary material for: Tailoring Polymeric Scaffolds with Buddleja globosa Extract for Dual Antimicrobial and Biocompatible Wound Healing Applications
Source: Molecules. 2025 May 31;30(11):2428. doi: 10.3390/molecules30112428 (PMC12155708; doi:10.3390/molecules30112428)
Supplement: Supplementary file 1 [file molecules-30-02428-s001.zip › Table S1 Supplementary.pdf]

Table S1. Vibrational assignment of scaffold S1* (without BG-126 extract).

**Frequency (cm^{-1})**	**Assignment**
1458	$\delta(CH)+\omega(CH_2)+\delta(OH)$
1383	$\delta(CH_2)+\delta(CH)+\delta(OH)$
1245	$\nu(C\text{-}C)+\nu(C\text{-}O)+\delta(CH)+\rho(CH_2)$
1162	$\nu(C\text{-}O\text{-}C)+\nu(\phi)$ main contributions
1108	
1041	$\rho(CH_3)+\delta(CH)+\delta(OH)$
944	$\nu(CN)$
863	$\rho(CH_2)+\nu(\phi)$
822	
