# Supplementary figures and images for: Tailoring Polymeric Scaffolds with Buddleja globosa Extract for Dual Antimicrobial and Biocompatible Wound Healing Applications
Source: Molecules. 2025 May 31;30(11):2428. doi: 10.3390/molecules30112428 (PMC12155708; doi:10.3390/molecules30112428)

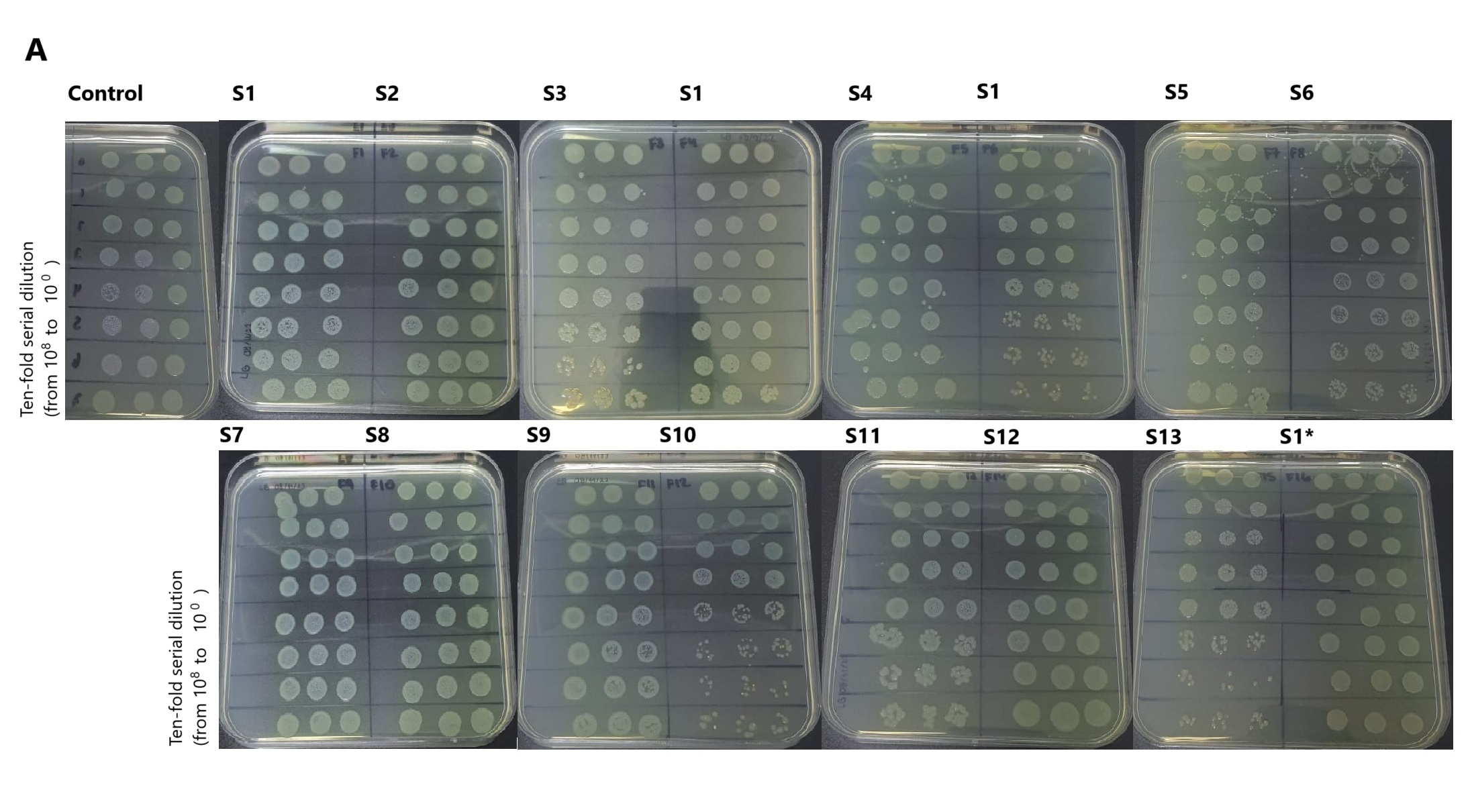

Supplement: Supplementary file 1 [file molecules-30-02428-s001.zip › Supp figure 1A.tif]

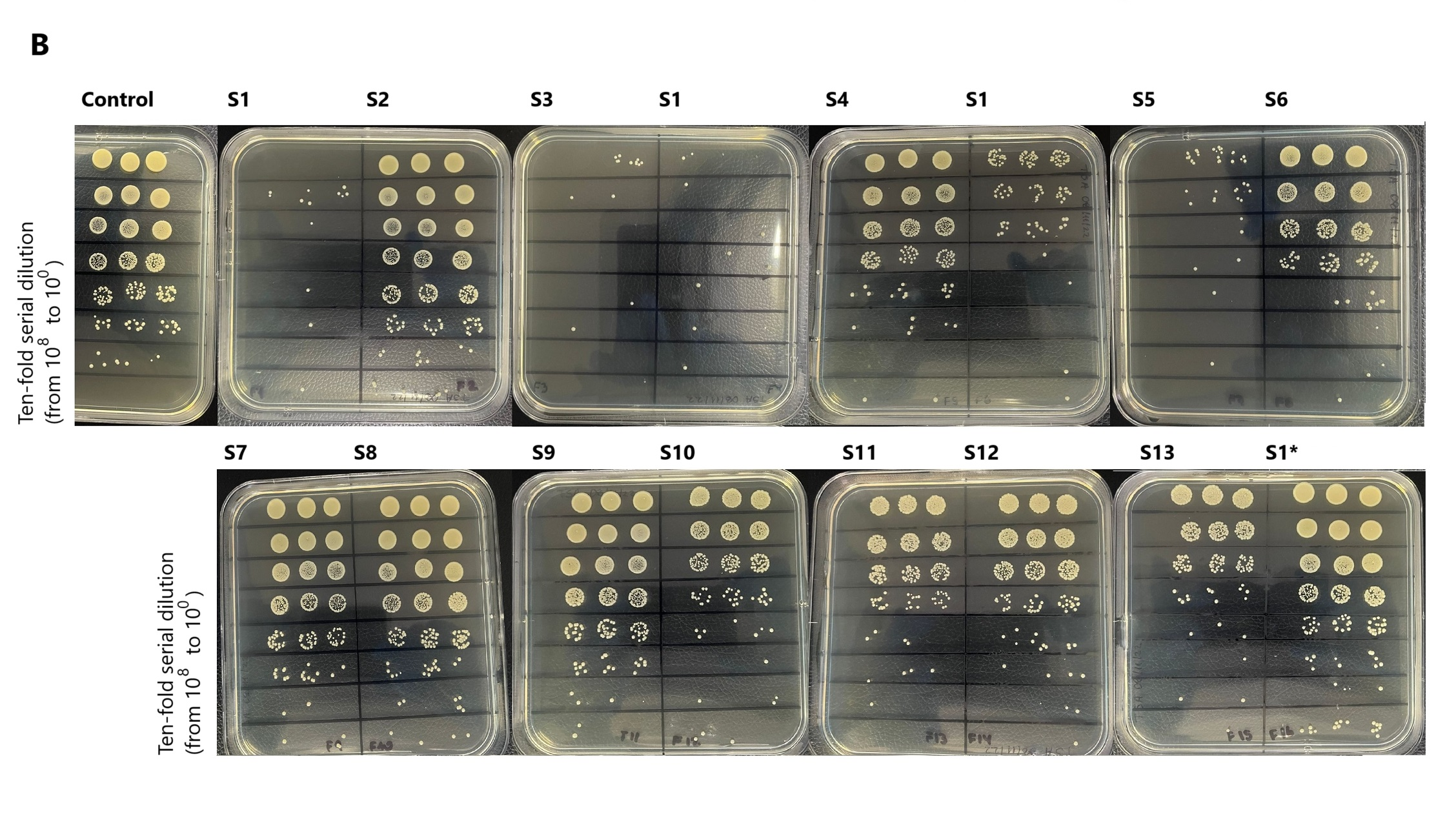

Supplement: Supplementary file 1 [file molecules-30-02428-s001.zip › Supp figure 1B.tif]
